# Supplementary material for: Material Design and Optimisation of Electrochemical Li-Ion Storage Properties of Ternary Silicon Oxycarbide/Graphite/Tin Nanocomposites
Source: Nanomaterials (Basel). 2022 Jan 26;12(3):410. doi: 10.3390/nano12030410 (PMC8838014; doi:10.3390/nano12030410)
Supplement: Supplementary file 1 [file nanomaterials-12-00410-s001.zip › nanomaterials-1555583 supplementary.pdf]

nanomaterials

Supplementary Materials

Material Design and Optimisation of Electrochemical Li-Ion Storage Properties of Ternary Silicon Oxycarbide/Graphite/Tin Nanocomposites

Dominik Knozowski [1], Pradeep Vallachira Warriam Sasikumar [2], Piotr Madajski [3], Gurdial Blugan [2], Maria Gazda [4], Natalia Kovalska [2] and Monika Wilamowska-Zawłocka [1,*]

[1] Department of Energy Conversion and Storage, Faculty of Chemistry, Gdańsk University of Technology, Narutowicza 11/12, 80-233 Gdańsk, Poland; dominik.knozowski@pg.edu.pl
[2] Laboratory for High Performance Ceramics, Empa, Swiss Federal Laboratories for Materials Science & Technology, CH-8600 Dübendorf, Switzerland; pradeep.variyar@gmail.com (P.V.W.S.); Gurdial.Blugan@empa.ch (G.B.); natalia.kovalska@empa.ch (N.K.)
[3] Faculty of Chemistry, Nicolaus Copernicus University in Torun, 87-100 Toruń, Poland; piotr.madajski@doktorant.umk.pl
[4] Department of Solid State Physics, Faculty of Applied Physics and Mathematics, Gdańsk University of Technology, Narutowicza 11/12, 80233 Gdańsk, Poland; margazda@pg.edu.pl
* Correspondence: monika.wilamowska@pg.edu.pl

Table S1. The results of deconvolution of Raman spectra for studied materials.

Material	D4		D1		D3		G		D2		I_{D1}/I_G	I_{D2}/I_G
	cm^{-1}	Int.	cm^{-1}	Int.	cm^{-1}	Int.	cm^{-1}	Int.	cm^{-1}	Int.		
**Graphite**	**1209**	0.054	1352	0.325	1515	0.103	1575	0.754	1603	0.193	0.431	0.256
SiOC	1210	0.100	1329	0.950	1510	0.223	1572	0.441	1605	0.379	2.154	0.859
SiOC/Sn-40%	1210	0.102	1345	0.881	1520	0.212	1577	0.451	1604	0.347	1.953	0.769
SiOC:C$_{0.2}$/Sn-40%	1260	0.036	1344	0.375	1520	0.112	1576	0.681	1603	0.244	0.551	0.323
SiOC:C$_{0.2}$/Sn-60%	1210	0.064	1342	0.380	1510	0.094	1570	0.790	1603	0.208	0.481	0.263
SiOC:C$_{0.1}$/Sn-60%	1214	0.045	1347	0.493	1519	0.124	1579	0.692	1608	0.351	0.712	0.507

Table S2. The results of deconvolution of Raman spectra for studied materials – continuation.

Material	D4		D1		D3		G		D2	
	cm^{-1}	Area. %	cm^{-1}	Area. %	cm^{-1}	Area. %	cm^{-1}	Area. %	cm^{-1}	Area. %
Graphite	1209	12.6	1352	38.5	1515	13.8	1575	19.6	1603	15.4
SiOC	1210	9.3	1329	63.2	1510	8.3	1572	12.5	1605	6.6
SiOC/Sn-40%	1210	7.8	1345	62.5	1520	8.7	1577	13.2	1604	7.8
SiOC:C$_{0.2}$/Sn-60%	1210	14.4	1342	41.7	1510	18.9	1570	21.4	1603	10.6
SiOC:C$_{0.2}$/Sn-40%	1260	3.0	1343	54.1	1520	11.8	1576	14.1	1603	16.9
SiOC:C$_{0.1}$/Sn -60%	1214	6.1	1347	57.3	1519	8.2	1579	14.1	1608	14.2

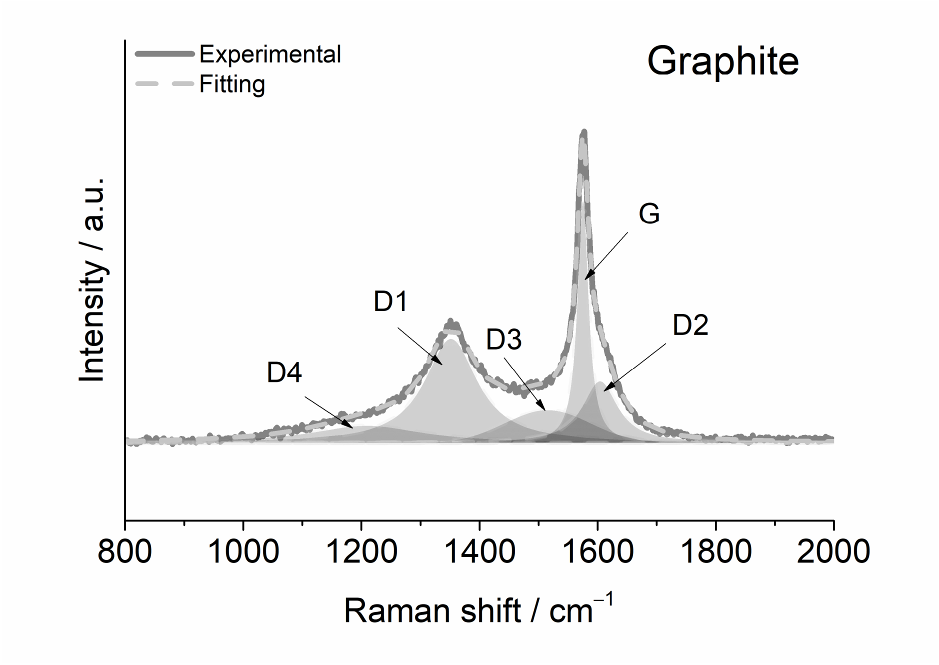

Figure S1. Raman fitting results for graphite.

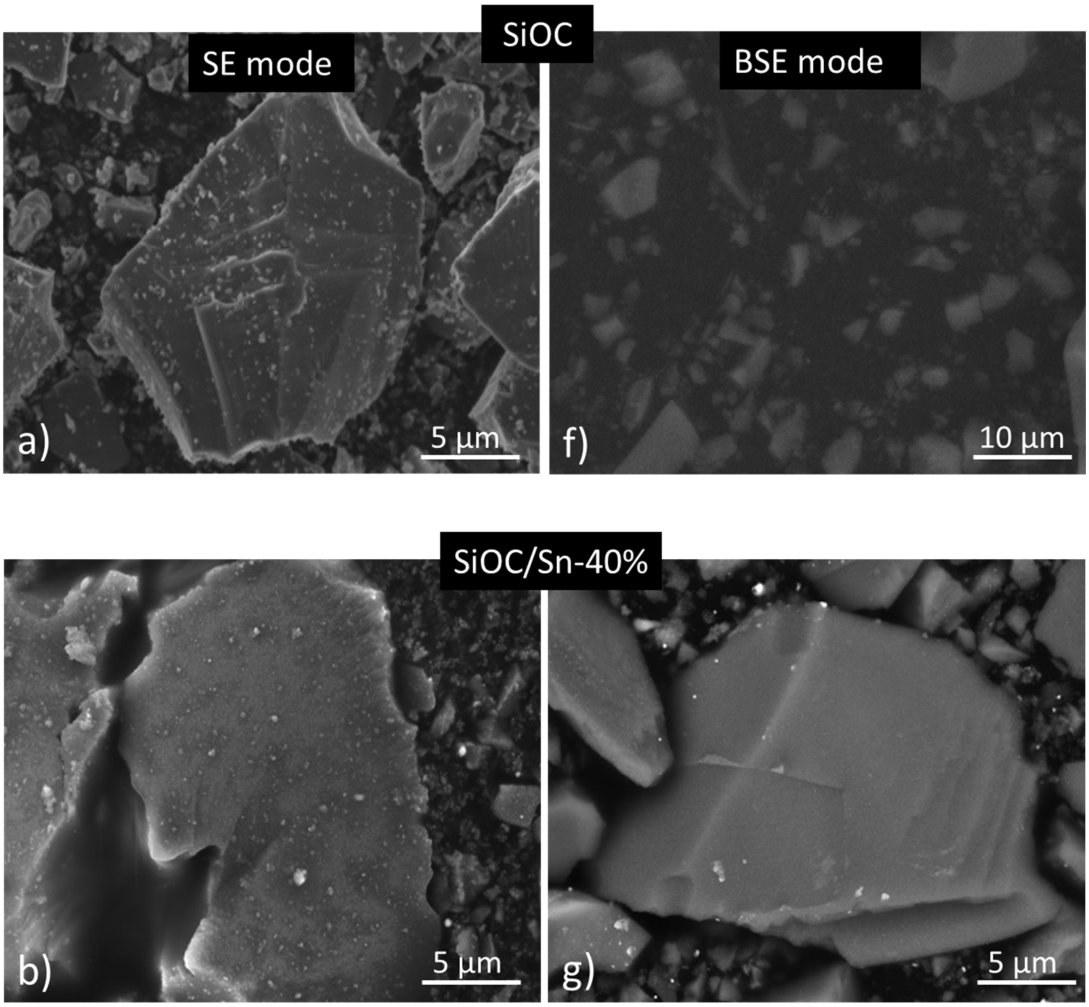

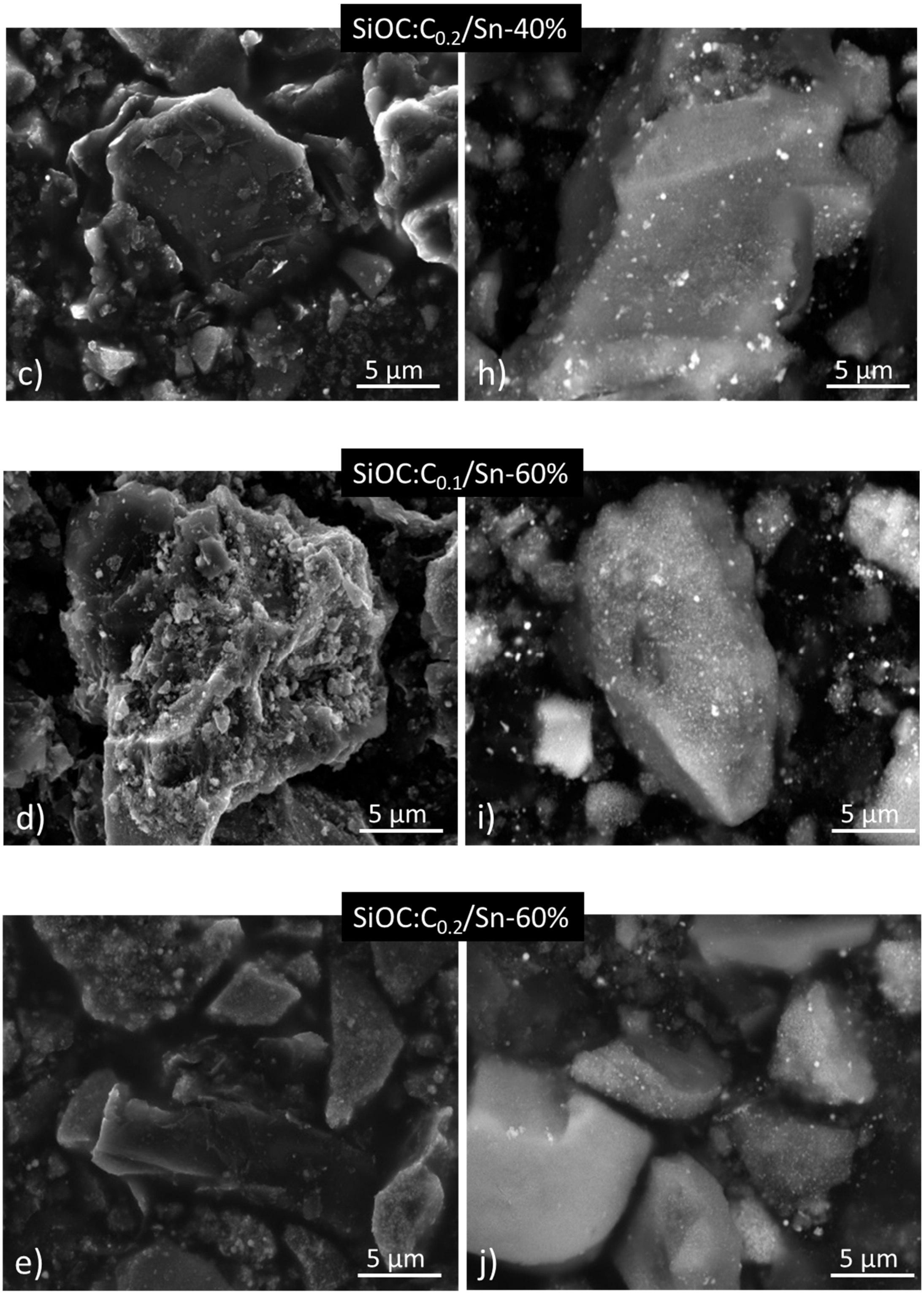

Figure S2. SEM images of SiOC, binary SiOC/Sn and ternary SiOC:C/Sn composites, (**a**–**e**) secondary electrons mode, (**f**–**j**) backscattering mode.

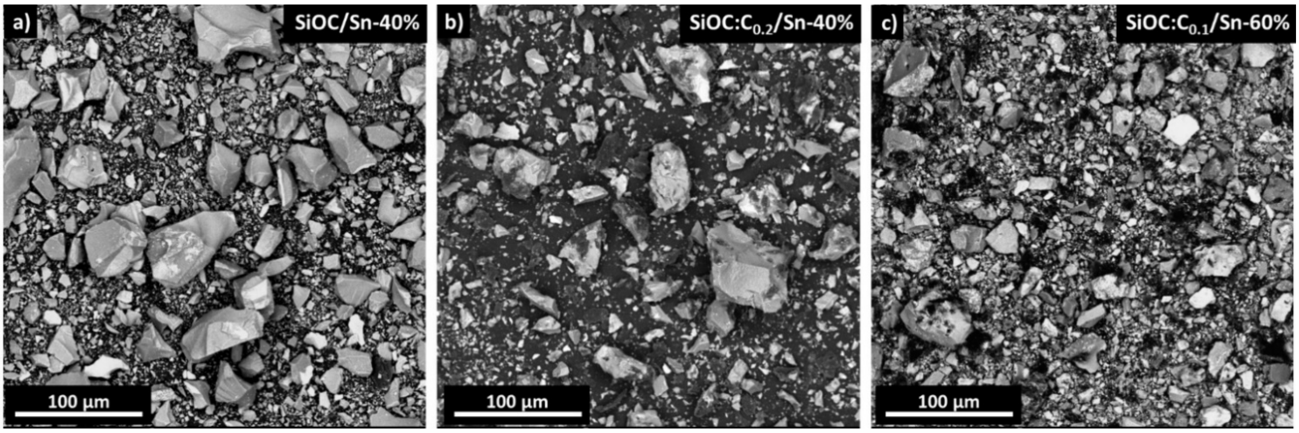

Figure S3. SEM images of (**a**) SiOC/Sn-40%, (**b**) SiOC:C0.2/Sn-40%, and (**c**) SiOC:C0.1/Sn-60% samples.

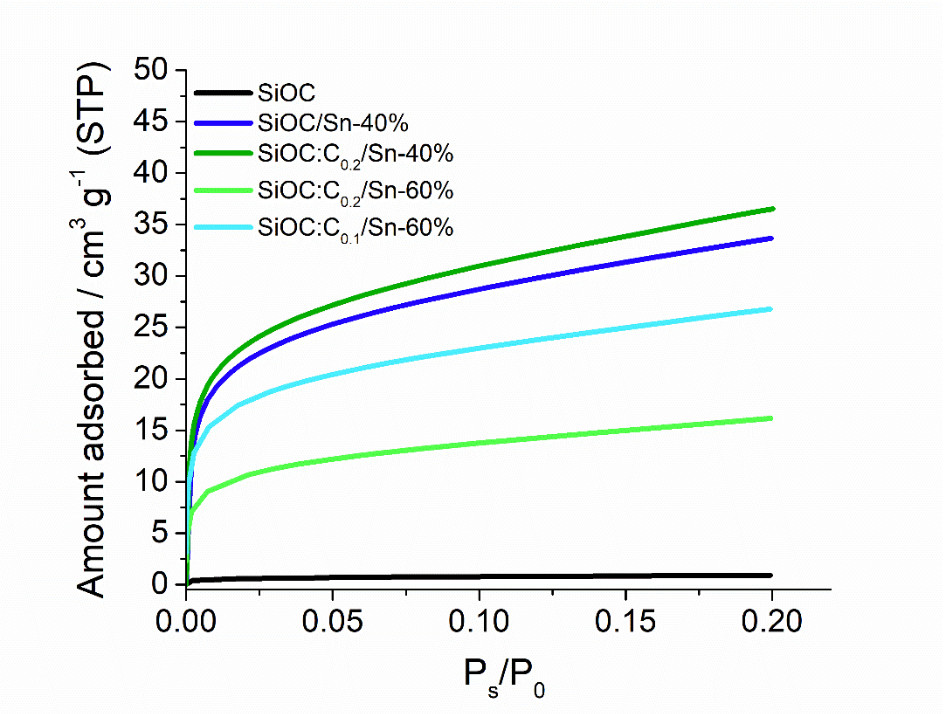

Figure S4. The adsorption curves of nitrogen (77 K) for obtained composite materials.

Table S3. BET surface area for obtained composite materials.

Sample composition	BET surface area [m³/g]
SiOC	3.18
SiOC/Sn-40%	118.2
SiOC:C0.2/Sn-40%	106.9
SiOC:C0.2/Sn-60%	55.1
SiOC:C0.1/Sn-60%	94.7

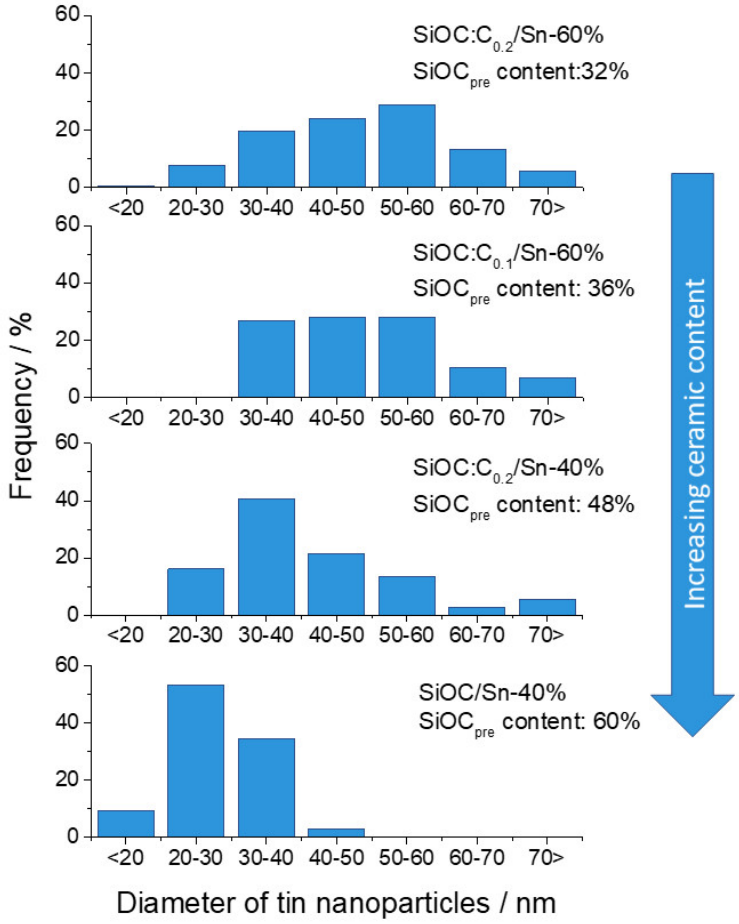

Figure S5. The size distribution of the Sn nanoparticles in the SiOC:C/Sn and SiOC/Sn nanocomposites. $SiOC_{Pre}$ content corresponds to the weight percentage content of preceramic polymer in preceramic blend.

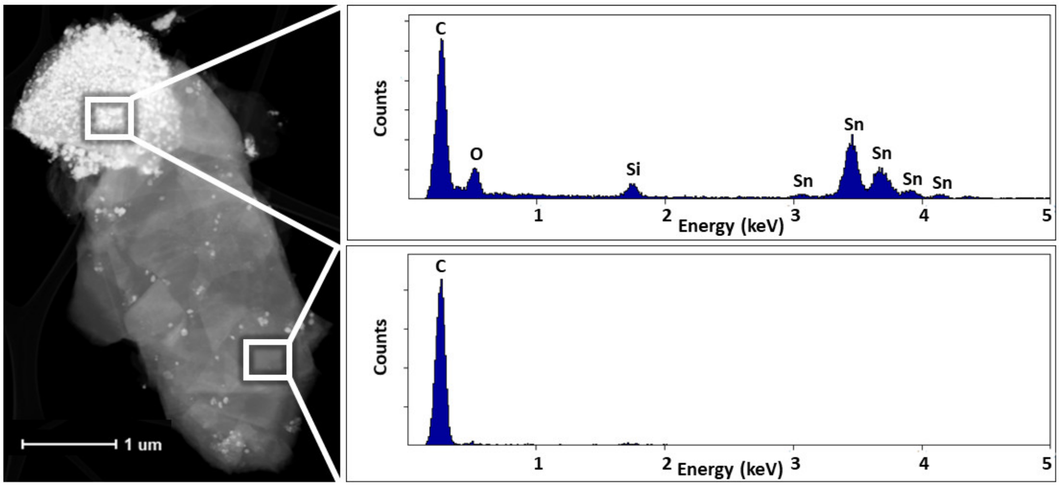

Figure S6. EDX elemental composition of tin-rich (SiOC) and tin-poor (graphite) regions of the SiOC:$C_{0.2}$/Sn-60% sample.

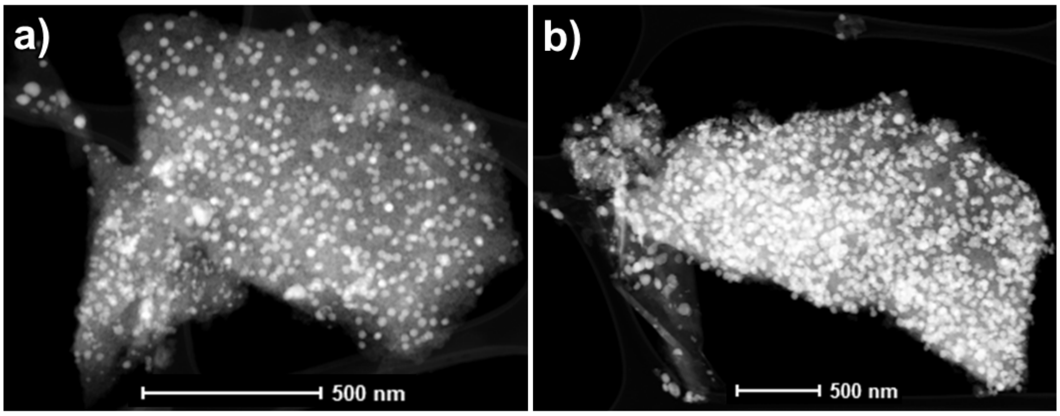

Figure S7. SEM images of (**a**) SiOC:C0.2/Sn-40% and (**b**) SiOC:C0.1/Sn-60% composites showing accumulation of tin nanoparticles in the ceramic phase.

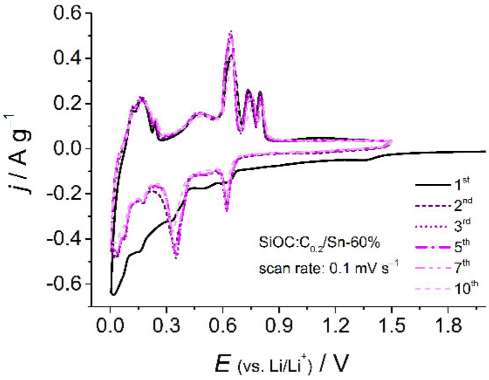

a)

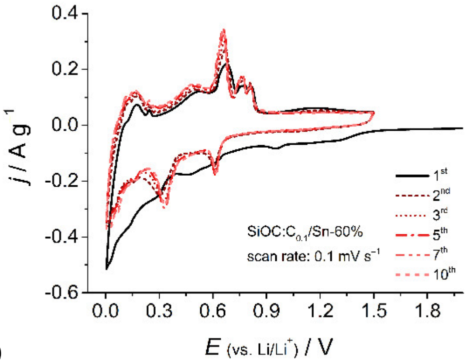

b)

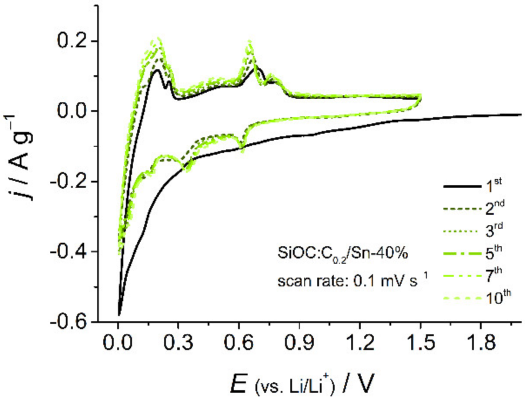

c)

Figure S8. Cyclic voltammetry curves of (**a**) SiOC:C0.2/Sn-60%, (**b**) SiOC:C0.1/Sn-60% and (**c**) SiOC:C0.2/Sn-40%.

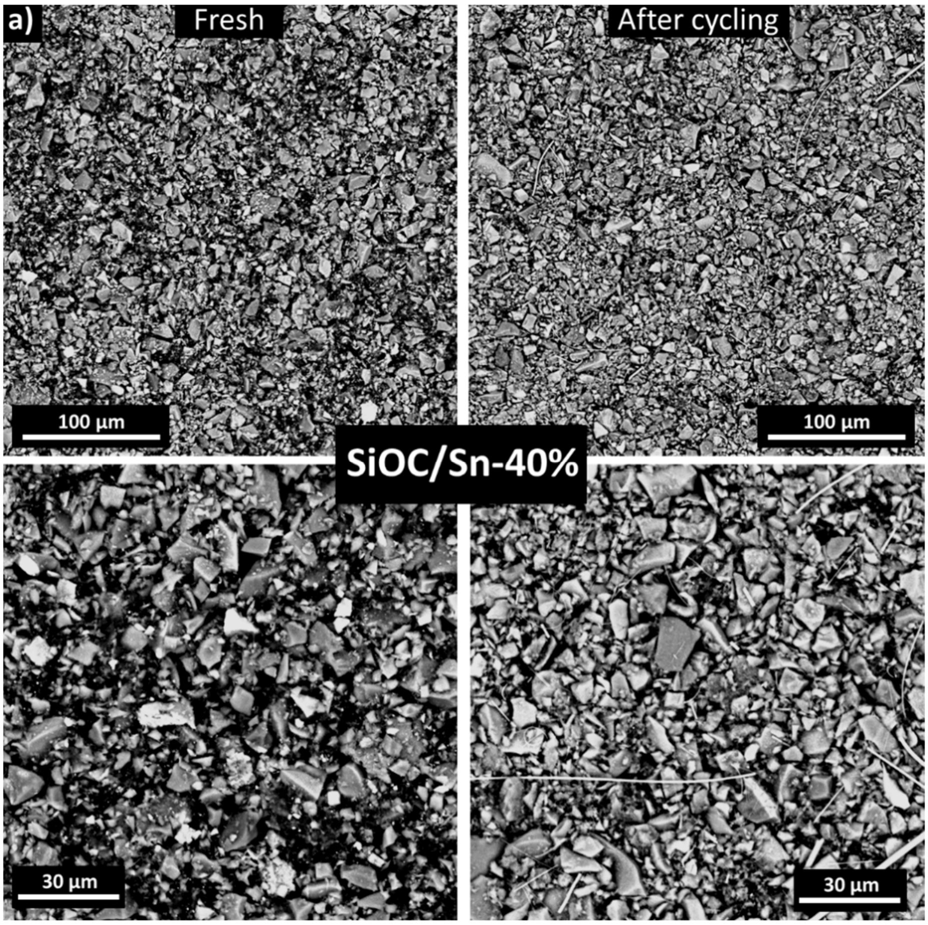
a)
Fresh
After cycling
100 µm
100 µm
SiOC/Sn-40%
30 µm
30 µm

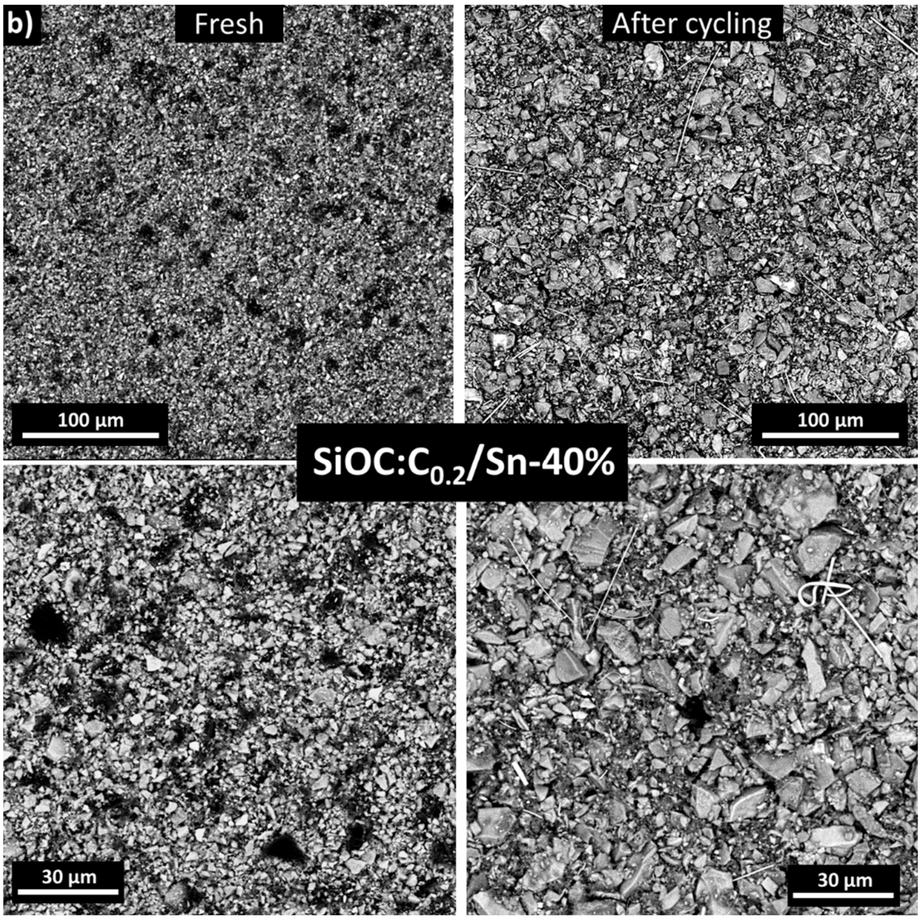

b)
Fresh
After cycling
100 µm
100 µm
SiOC:C0.2/Sn-40%
30 µm
30 µm

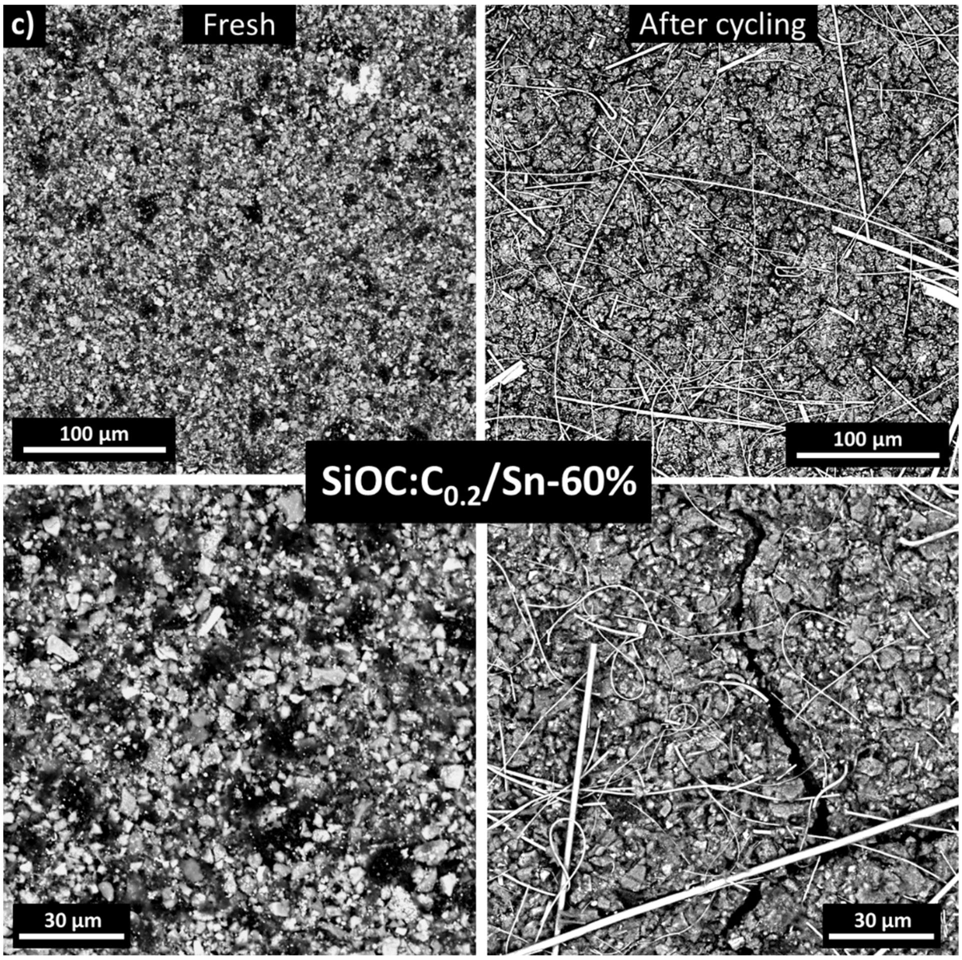

Figure S9. SEM images of electrode layers before ("fresh") and after extended cycling tests (2 cycles at 18.6 mA g^{-1} followed by 100 cycles at 372 mA g^{-1}) recorded for (**a**) SiOC/Sn-40%, (**b**) SiOC:C$_{0.2}$/Sn-40% and (**c**) SiOC:C$_{0.2}$/Sn-60%.

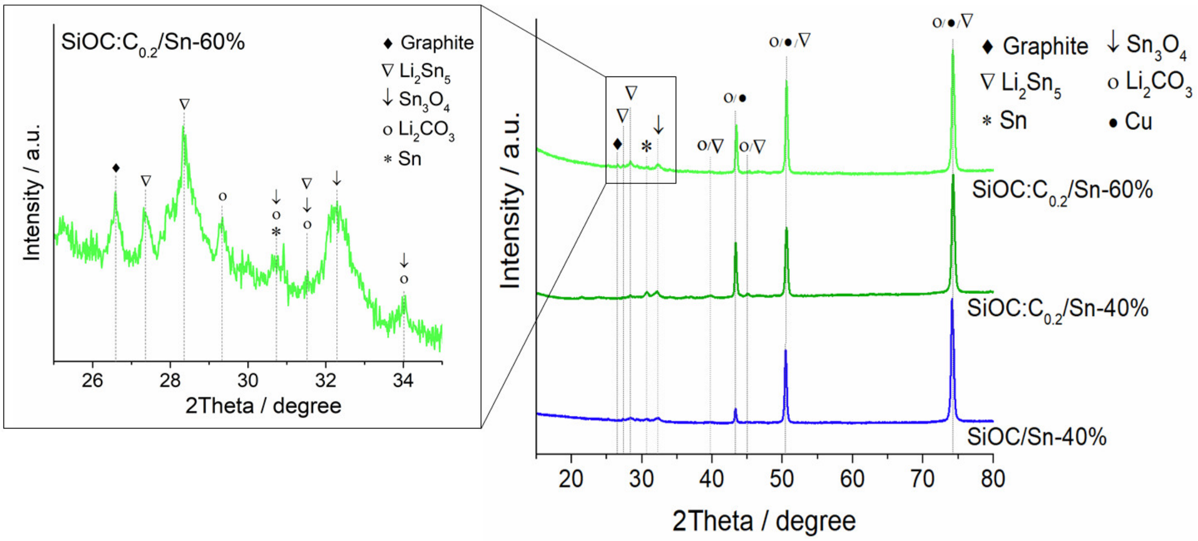

Figure S10. Diffractograms of the cycled electrodes, Inset: the selected 2Theta range of the diffractogram of SiOC:C$_{0.2}$/Sn-60% composite.

Table S4. Data obtained from XRD according to PDF card Sn-ref_00-004-0673.

**Name and formula**	
Reference code:	00-004-0673
Mineral name:	Tin, syn
PDF index name:	Tin
Empirical formula:	Sn
Chemical formula:	Sn
**Crystallographic parameters**	
Crystal system:	Tetragonal
Space group:	I41/amd
Space group number:	141
a (Å):	5.8310
b (Å):	5.8310
c (Å):	3.1820
Alpha (°):	90.0000
Beta (°):	90.0000
Gamma (°):	90.0000
Calculated density (g/cm^3):	7.29
Volume of cell (10^6 pm^3):	108.19
Z:	4.00
RIR:	1.97
**Subfiles and Quality**	
Subfiles:	Inorganic<br>Mineral<br>Alloy, metal or intermetalic<br>Common Phase<br>Educational pattern<br>Forensic<br>NBS pattern
Quality:	Star (S)
**Comments**	
Color:	Light gray metallic
General comments:	Space group given by Mark, Polanyi, Z. *Phys.*, **18** 75-96 (1925).<br>Color from *Dana's System of Mineralogy, 7th Ed.*, 485.
Sample source:	Sample was furnished by Johnson Matthey Company, Ltd. and annealed

	12 hours at 160 C.					
Analysis:	Analysis (wt.%): Pb 0.0012, Sb 0.001, Fe 0.00027, Cu 0.0002, As 0.0002, Bi 0.00012, S 0.0003, Sn 99.997 (by difference), other form a-Sn (cubic).					
Additional pattern:	See ICSD 40037 (PDF 86-2264).					
Melting point:	231.8°					
Temperature:	Pattern taken at 26 C					
**Peak list**						
No	h	k	l	d [A]	2Theta[deg] I [%]	
1	2	0	0	2.91500	30.645	100.0
2	1	0	1	2.97300	32.019	90.0
3	2	2	0	2.06200	43.872	34.0
4	2	1	1	2.01700	44.903	74.0
5	3	0	1	1.65900	55.332	17.0
6	1	1	2	1.48400	62.540	23.0
7	4	0	0	1.45800	63.785	13.0
8	3	2	1	1.44200	64.578	20.0
9	4	2	0	1.30400	72.416	15.0
10	4	1	1	1.29200	73.198	15.0
11	3	1	2	1.20500	79.472	20.0
12	4	3	1	1.09500	89.412	13.0
13	1	0	3	1.04340	95.167	3.0
14	3	3	2	1.04010	95.565	5.0
15	4	4	0	1.03090	96.699	2.0
16	5	2	1	1.02520	97.418	5.0
17	2	1	3	0.98240	103.275	5.0
18	6	0	0	0.97180	104.868	2.0
19	3	0	3	0.93100	111.663	3.0
20	5	1	2	0.92860	112.101	13.0
21	6	2	0	0.92190	113.348	5.0
22	6	1	1	0.91780	114.130	5.0
23	3	2	3	0.88680	120.599	4.0
24	5	4	1	0.87550	123.246	2.0
25	4	1	3	0.84850	130.416	4.0
26	5	3	2	0.84660	130.976	10.0
27	6	3	1	0.83860	133.431	4.0
28	6	4	0	0.80860	144.590	6.0

29	7	0	1	0.80580	145.859	3.0

Stick Pattern

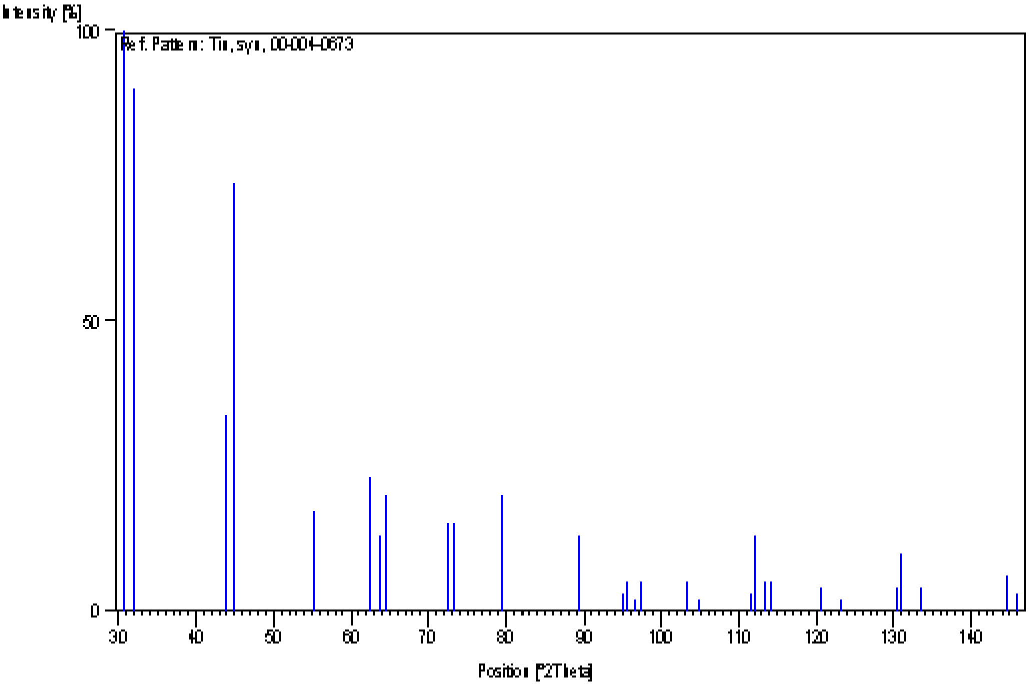

References

1. Swanson, T. *Natl. Bur. Stand. (U.S.), Circ.* **1953**, *539 I*, 24.
